# Developmental trauma disorder: pros and cons of including formal criteria in the psychiatric diagnostic systems

**DOI:** 10.1186/1471-244X-13-3

**Published:** 2013-01-03

**Authors:** Marc Schmid, Franz Petermann, Joerg M Fegert

**Affiliations:** 1 Department of child and adolescent psychiatry University Basel, Schanzenstrasse 13, CH-4056, Basel, Switzerland; 2 Center of clinical psychology and rehabilitation University Bremen, Grazer Strasse 6, DE-28329, Bremen, Germany; 3 Department of Child and Adolescent Psychiatry and Psychotherapy, University of Ulm, Steinhövelstrasse 5, DE-89075, Ulm, Germany

**Keywords:** Comorbidity, Developmental psychopathology, Developmental trauma disorder (DTD), Dissociation, Post-traumatic stress disorder (PTSD)

## Abstract

**Background:**

This article reviews the current debate on developmental trauma disorder (DTD) with respect to formalizing its diagnostic criteria. Victims of abuse, neglect, and maltreatment in childhood often develop a wide range of age-dependent psychopathologies with various mental comorbidities. The supporters of a formal DTD diagnosis argue that post-traumatic stress disorder (PTSD) does not cover all consequences of severe and complex traumatization in childhood.

**Discussion:**

Traumatized individuals are difficult to treat, but clinical experience has shown that they tend to benefit from specific trauma therapy. A main argument against inclusion of formal DTD criteria into existing diagnostic systems is that emphasis on the etiology of the disorder might force current diagnostic systems to deviate from their purely descriptive nature. Furthermore, comorbidities and biological aspects of the disorder may be underdiagnosed using the DTD criteria.

**Summary:**

Here, we discuss arguments for and against the proposal of DTD criteria and address implications and consequences for the clinical practice.

## Background

Inclusion of post-traumatic stress disorder (PTSD) in psychiatric diagnostic systems represents an important milestone since a clear connection between traumatic experiences and mental disorders have not been established previously [1-3]. Clinicians in the field of child and adolescent psychiatry and clinical psychology have to face acute traumatized children and victims of different shades and forms of chronic child abuse, maltreatment and neglect.

In the clinical setting, the effects of neglect, maltreatment, and abuse are noticeable which has prompted the need for a diagnosis capable of creating the connection between developmental and psychopathological aspects.

In children and adolescents, the usefulness of diagnostic criteria of PTSD is limited because the characterization of the condition is based on symptoms in adults. Because most symptoms are subjective and require verbal description by the patient, the diagnosis of PTSD in younger children remains challenging. In the presence of distinct, well-defined traumata and their effects, the diagnosis of PTSD can be readily made; childhood traumatization and neglect tend to be more complex and may entail a multitude of psychosocial risk factors. Therefore, various proposals for diagnostic criteria have been published which include developmental psychology factors [4-7].

Most traumatic experiences in children and adolescents occur in their immediate social environment [5,8,9]. Families with neglected, maltreated, or abused children often carry a number of additional risk factors, such as mental disorders in parents, poverty, cramped living conditions, or social isolation [5,10,11]. Moreover, childhood traumatization leads to a significantly higher risk of suffering other traumata in adult life [12,13].

Many severely maltreated, sexually abused, or neglected children who had suffered repeated traumatic events (i.e., chronic or sequential traumatization) do not fulfill the diagnostic criteria of PTSD in the strict (adult) sense. Frequently, affected children experience a multitude of other psychopathological symptoms [14-16] that often persist into adulthood, thus making a more systematic description of the particular symptoms necessary. Terr’s concept [2], one of the most influential proposals for the improvement of diagnostic processes, categorized traumata into single, well-defined, more public traumata such as accidents, natural disasters, and wartime experiences (type I), and a series of related, sequential traumata such as neglect, maltreatment, and sexual abuse often committed secretly and over longer time periods by persons close to the victim (type II). While type I traumatization often produces the classic psychopathological symptoms of PTSD, sequential traumatization may result in impaired development of personality and heterogeneous psychopathological symptoms. Dissociation, low self-efficacy, impaired regulation of emotion, somatization, and disturbed perception of self and others are all among the symptoms caused by chronic traumatization [2].

**Figure 1:**
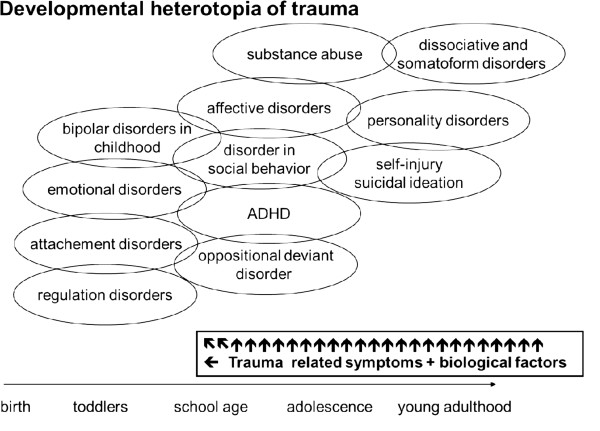
Development heterotopia of trauma.

Repeatedly traumatized patients tend to exhibit a typical pattern of successive disorders, i.e., regulatory disorder during infancy, attachment disorders with or without disinhibition at preschool age, hyperkinetic conduct disorder at school age, or combined conduct and emotional disorders during adolescence. In later years, personality disorders are common and often accompanied by substance abuse, self-harm, and affective disorders. It is assumed that the same fundamental deficiencies (like impaired regulation of emotion, low self-efficacy, tendency towards dissociation) have variable consequences at different developmental stages of the patient, thus resulting in typical age-related psychopathological symptoms [17] (see Figure 1).

Most literature reviews in this field focus on cross-sectional studies. Longitudinal studies are rare as they are difficult to conduct and constrained by ethical limitations. There are only a few highly important studies supporting the relevance of interpersonal trauma for developmental psychopathology from childhood to adulthood [4,14,18].

Empirical evidence of the course of PTSD indicates that severe sequential traumatization mostly begins in childhood showing an inverse correlation between the age of onset of traumatization and the severity of symptoms. This gave rise to the need of improved understanding of developmental aspects in children and adolescents with a complex trauma history [9].

In an effort to establish a rational diagnosis in severely traumatized children, several authors postulated a refined list of criteria [1,17,19]. To separate these criteria from those for PTSD, the term ‘developmental trauma disorder’ (DTD) was suggested [20] (see list of symptoms below).

### List of symptoms: consensus of proposed diagnostic criteria for developmental trauma disorder

In the present paper, the suitability and limitations of the criteria postulated in the diagnosis of DTD are reviewed, and implications and consequences for clinical practice are discussed.

### Proposed diagnostic criteria and symptom clusters

To include DTD in the DSM-V algorithm for separated diagnosis, van der Kolk *et al.*[20] proposed the following criteria (organized into three symptom clusters) in addition to the defined symptoms of PTSD (see List of Symptoms below):

· Symptoms of emotional and physiological dysregulation/dissociation

· Problems with conduct and attention regulation

· Difficulties with self-esteem regulation and in managing social connections.

In the following, these symptom clusters are addressed in more detail.

### Symptoms of emotional and physiological dysregulation/dissociation

Chronic activation of neurobiological systems involved in the regulation of stress and emotion appears to potentiate activation of the relevant neurotransmitters and neuroendocrinological systems. This has also been implicated in severe emotional dysregulation [21,22]. Several studies reported clear differences in the aptitude of children with and without traumata in regulation and recognition of emotion [23-25].

Subjects with difficulties in regulation of emotion react faster and more fiercely to emotional stimuli and require more time to calm down after an emotional reaction. This was particularly evident in studies with adult borderline patients [26-28]. Moreover, negative emotional reactions in everyday life seem to be more easily triggered in those patients [29,30].

### A. Exposure

The child or adolescent has experienced or witnessed multiple or prolonged extremely stressful traumatic events over a period of at least one year beginning in childhood or early adolescence, including:

1) Direct experience or witnessing of repeated and severe episodes of interpersonal violence, and

2) Significant disruptions of protective care giving as a result of repeated changes in primary caregiver, repeated separation from the primary caregiver, or exposure to severe and persistent emotional abuse.

### B. Affective and physiological dysregulation

The child exhibits impaired normative developmental competencies related to arousal regulation, including at least two of the following:

1) Inability to modulate, tolerate, or recover from extreme affect states (e.g. fear, anger, shame), including prolonged and extreme tantrums, or immobilization,

2) Disturbances in regulation of bodily functions (e.g. persistent disturbances in sleeping, eating, and elimination; over-reactivity or under-reactivity to touch and sounds; disorganization during routine transitions),

3) Diminished awareness/dissociation of sensations, emotions, and bodily states, and/or

4) Impaired capacity to describe emotions or bodily states.

### C. Attentional and behavioral dysregulation

The child exhibits impaired normative developmental competencies related to sustained attention, learning, or coping with stress, including at least three of the following:

1) Preoccupation with threat or impaired capacity to perceive threat, including misreading of safety and danger cues,

2) Impaired capacity for self-protection, including extreme risk-taking or thrill-seeking,

3) Maladaptive attempts at self-soothing (e.g. rocking and other rhythmical movements, compulsive masturbation),

4) Habitual (intentional or automatic) or reactive self-harm, and/or

5) Inability to initiate or sustain goal-directed behavior.

### D. Self and relational deregulation

The child exhibits impaired normative developmental competencies in his/her sense of personal identity and involvement in relationships, including at least three of the following:

1) Intense preoccupation with safety of the caregiver or other loved ones (including precocious care giving) or difficulty tolerating reunion with them after separation,

2) Persistent negative sense of self, including self-loathing, helplessness, worthlessness, ineffectiveness, or defectiveness,

3) Extreme and persistent distrust, defiance or lack of reciprocal behavior in close relationships with adults or peers,

4) Reactive physical or verbal aggression toward peers, caregivers, or other adults,

5) Inappropriate (excessive or promiscuous) attempts to achieve intimate contact (including but not limited to sexual or physical intimacy), or excessive reliance on peers or adults for safety and reassurance, and/or

6) Impaired capacity to regulate empathic arousal as evidenced by lack of empathy for, or intolerance of, expressions of distress of others, or excessive responsiveness to the distress of others.

### E. Post-traumatic spectrum symptoms

The child exhibits at least one symptom in at least two of the three PTSD symptom clusters B, C, and D.

### F. Duration of disturbance

Persistence of symptoms in criteria B, C, D, and E for at least 6 months.

### G. Functional impairment

The disturbance causes clinically significant distress or impairment in at least two of the following areas of functioning:

1) *Scholastic*: under-performance, non-attendance, disciplinary problems, drop-out, failure to complete degree/credential(s), conflict with school personnel, learning disabilities, or intellectual impairment that cannot be accounted for by neurological or other factors,

2) *Familial*: conflict, avoidance/passivity, running away, detachment and surrogate replacements, attempts to physically or emotionally hurt family members, non-fulfillment of responsibilities within the family,

3) *Peer group*: isolation, deviant affiliations, persistent physical or emotional conflict, avoidance/passivity, involvement in violence or unsafe acts, age-inappropriate affiliations or style of interaction,

4) *Legal*: arrests/recidivism, detention, convictions, incarceration, violation of probation or other court orders, increasingly severe offenses, crimes against other persons, disregard or contempt for the law or for conventional moral standards, and/or

5) *Health*: physical illness or problems that cannot be fully accounted for, physical injury or degeneration, involving the digestive, neurological (including conversion symptoms and analgesia), sexual, immune, cardiopulmonary, proprioceptive, or sensory systems, severe headache (including migraine), or chronic pain or fatigue.

Dissociation may be described as a loss of outward perception and trance-like state of mind, which is accompanied by a loss of coenesthesia and sense of time, spatial orientation, facial expression, perception of pain, and often a feeling of derealization. Dissociative disruptions may also involve the loss of memory of own and observed external actions. As shown in recent experiments [31], both learning and assimilation of new information are strongly inhibited in dissociated states. Lynch *et al.*[32] demonstrated that reducing dissociation tendency improves the success of outpatient psychotherapy.

While approximately 10% of the general population reacts with a stronger tendency to dissociation in response to trauma, 50% of affected individuals may suffer from chronic dissociation when faced with repeated traumatization [33,34]. Apart from the genetic disposition, susceptibility of reacting to traumatic experiences with dissociation is markedly influenced by the frequency and nature of traumatic experiences. Furthermore, dissociation tendency is a predictor for the development of PTSD in response to traumatic experiences [35,36].

Maltreated or sexually abused schoolchildren have a much stronger tendency towards dissociation than non-maltreated children [37]. Extreme familial psychosocial stress and a tense family atmosphere are both factors that appear to potentiate this tendency [38,39].

### Somatization, body and sensory perception

Among chronically traumatized individuals, body perception is frequently impaired [40]. Good body perception is necessary for recognizing, processing, and expressing emotions [41]. In traumatized individuals, perception of pain during tense conditions is diminished [42,43], and auditory perception is impaired [44]. Overall, body perception, sensory perception, experience of pleasure, and ability to focus on positive sensory perceptions such as taste and music are clearly underdeveloped in affected individuals.

Studies show a clear relationship between early experiences of neglect/malnutrition and somatic diseases (e.g. high blood pressure, coronary heart disease, diabetes) in adulthood [45]. Furthermore, there is increasing evidence that PTSD is not only associated with a higher vulnerability for comorbid mental disorders but also with an increased incidence of (psycho-) somatic disorders [46,47]. Many traumatized children suffer from severe sleep disorders [48,49].

### Self-injury, high risk behavior, and sexual abnormalities

Non-suicidal self-injury [50] and suicidal behavior [51,52] constitute the symptoms most strongly linked with traumatization, particularly sexual abuse. More than 80% of patients with a history of self-injury report traumatic events in their earlier lives [53]. Given the high prevalence of self-injuries among adolescents, periodical and repetitive self-injuring behavior should be regarded separately since it is unlikely that the majority of adolescent self-injurers share a history of traumatic events. Interestingly, repetitive self-injury has been reported more often in adults with a childhood history of sexual abuse, whereas intermittent self-injury appears to be more frequently associated with physical abuse in childhood [53]. Nonetheless, a meta-analysis of 45 studies on the association of sexual abuse and self-injury only found a relatively weak relationship between self-injury and sexual abuse indicating that sexual abuse ceased to explain the variance in self-injurious behavior if the studies were controlled for other psychiatric risk factors [54]. Other studies described a relationship between self-injury and traumatization [55,56]. A recent review suggests that the association of child maltreatment and self-injury varies according to the type of maltreatment [57]. Weierich and Nock showed that PTSD symptoms mediate the relation between sexual abuse and self-injury [55]. Self-injury probably functions as a support of emotion regulation and disrupts dissociative states and the emotional tension related to regulation of emotion [58]. Neurobiologically, self-injury can be seen as an attempt to alter the state of the autonomic nervous system that has been pushed to an extreme state by reminders of traumatic events [59].

Glassman *et al*. [60] found that traumatization leads to self-injury, particularly when shame and self-criticism transform into self-hate. Among all psychological disorders, post-traumatic syndromes are most closely related to suicidal ideation and are associated with the highest suicide rates. PTSD symptoms like flashbacks, nightmares and intrusions were reported to be significantly associated with tension, dissociation and self-injury [53,58]. The literature concerning the association between a history of traumatic events and suicidal behavior is particularly consistent. Recent data from the World Mental Health survey (21 countries, n=55’299) showed a strong relationship between childhood adversities (odds ratio [OR] for suicide attempt after sexual abuse: 5.7) and suicidal behavior, such as suicidal ideation or attempts [51].

Children who have experienced sexual abuse seem to be more preoccupied with their sexuality, show more sexualized behavior, and may exhibit compulsive masturbating behavior [61,62]. Several reviews suggest that impulsive high-risk-behavior in adolescents (e.g. unprotected sexual intercourse, risky behavior in traffic, carrying arms) often occurs in young individuals who have been traumatized [63]. In particular, early substance abuse is likely alongside impulsivity and psychosocial risk factors [64].

### Difficulties with executive functions and the regulation of attention

Studies in heavily deprived Romanian orphans showed that without a minimum of stimulation during the sensitive phase of development, cognitive development is sustainably impaired [65-67]. Executive functions, such as attention span, distractibility, and the ability for serial structuring and making plans are particularly affected. However, there is a clear distinction between these traits and the symptoms of attention deficit hyperactivity disorder (ADHD) [67,68]. The work group around Michael Rutter analyzed the intelligence profiles of traumatized and neglected Romanian residential care children after adoption by families in the United Kingdom and noticed that these children show deficits in their executive functions [65,68]. On the neuropsychological level, self-regulation of more complex behaviors and future orientated planning in daily life appear to be limited or impaired, because complex traumatized children have learned to focus on the next moment to survive and not to overlook broader timeframes [17,19]. Some studies showed different significant problems in working memory in students following sexual abuse and childhood trauma [69,70]. Endo and colleagues [71] found that dissociative children meet criteria of ADHD whereas non maltreated children with ADHD do not show dissociative symptoms.

### Difficulties in self-regulation and establishment of relationships

Eighty percent of all traumatized (physically abused) children show a disorganized style of attachment [72-74]. Abused and neglected children often develop highly insecure representations of attachment [75,76] and show promiscuous and non-selective behavior in their attachment to adults [67,77]. Other studies found that exposure to interpersonal trauma leeds to social isolation [78]. Attachment is an important resilience factor for preventing the development of a mental disorder after traumatization. Emotional support provided directly following a traumatizing experience reduces the risk of developing PTSD [35,36]. Moreover, positive relationships enhance the success of psychosocial interventions [79].

Perception of social situations is altered in traumatized children because they are highly sensitized to potentially threatening stimuli. Dodge and Schwartz [80] showed that traumatized children tend to interpret neutral behavior of other people as hostile and react to more aggressive behavior with fear or even dissociation. Furthermore, abused children react stronger and more impulsive to negative facial expressions, especially to those of anger [23,81-83]. This hypersensitivity to potentially threatening stimuli often leads to aggressive reactions in affected individuals [80,84,85]. Probably reduced grey matter in the visual cortex represents a neurobiological correlate of difficulties in the recognition and interpretation of emotions and social skills [86].

Traumatized individuals often develop feelings of self-reproach, guilt, and shame [87]. Development of a healthy self-image is substantially impaired in traumatized subjects. The impact of abuse and neglect on the development of self-esteem (self-insufficiency, defectiveness) has been addressed in longitudinal studies [85,88]. Kim and Cicchetti reported that feelings of shame caused by traumatization were responsible for interpersonal problems in adulthood [88].

Several studies have addressed empathy, theory of mind, and the ability for mentalization in traumatized and heavily neglected children [89,90]. The ability of taking the perspectives of others was diminished, increasing with the length of the children being in conditions of deprivation [66]. When studying mentalization [91], the ability to take the perspective of others in emotional situations involving pressure was of main interest. Under such circumstances, deficient regulation of emotion and lacking ability to take others perspectives are additive.

## Discussion

Because of scientific discussion among the long term consequences of childhood trauma and the criteria of development trauma disorders a discussion among pros and cons of the introduction of such a diagnosis in the revision of DSM-V and ICD-11 started (see Table 1).

**Table 1:** Arguments for and against the introduction of development trauma disorder in the psychiatric diagnostic systems

**Arguments: pro development trauma disorder**	**Arguments against development trauma disorder**
Specific diagnosis for observed symptoms from severely traumatized children	Conflicts the traditional diagnostic systems on constraining on the description of symptoms
Regards developmental psychopathology and the course of mental disorders	Assumed mono-causality is conflicting bio-psycho-social model of the etiology of mental disorders
Explains co-morbidity	Underestimates the aspects of inverse correlations of psychopathology and traumatization
Enables effective treatment for co-morbid disorders	Selectivity underestimates the role of resilience
Enhances research in the field of developmental psychopathology and trauma related disorders	Higher risk to miss co-morbid disorders and effective (psycho-) pharmaceutical treatment
Show scientific based arguments for a improvement of child protection, prevention and resources of youth welfare services	Failed to define age-related symptoms
Explains severe problem behavior, for example reactive aggression, chronic dissociation and self-injury	Trauma focused explorations might lead to a problem focused exploration style

### Arguments for and against a systematic diagnosis of DTD

#### Arguments in favor of formalized DTD diagnostic criteria

The following arguments support the initiative to include DTD as a distinct mental disorder in diagnostic systems:

*· More specific diagnosis*: The diagnosis of PTSD does not sufficiently take into account the symptoms of traumatized patients. The postulated DTD diagnostic criteria comprise a range of symptoms seen to occur after complex and repeated traumatization. For the diagnosis of DTD, traumatic experience is essential but not exclusive, and genetic and biopsychosocial origins of the disorder must be ruled out to specify the interaction between neurobiology, epigenetics and transgenerational traumatic life events and their consequenses for the development of mental disorders. The existence of specific and validated DTD diagnostic criteria may sensitize professionals and the general public to the drastic consequences of child abuse, neglect, and traumatization. Moreover, the establishment of measures for e.g. child protection, policy making would be expedited.

*· Course of mental disorders*: The supporters of this initiative argue that more emphasis should be placed on developmental aspects of disorders caused by traumatization. The few longitudinal studies available indicate that more than 60% of adults with psychiatric disorders suffered from psychopathological symptoms during adolescence, and 77% exhibited symptoms before the age of 18 years [87,92]. Furthermore, PTSD frequently becomes chronic. In a longitudinal study in adolescents with PTSD, 48% of patients still met the criteria for PTSD three to four years later [93].

*· Enhance research*: Establishment of formal diagnostic criteria for DTD is expected to stimulate research efforts in this area (e.g., epidemiological studies, developmental-psychopathological research). Cross-sectional and longitudinal studies on psychosocial risks and comorbidities during childhood and adolescence should be encouraged.

*· Explain comorbidities*: From a clinical point of view, the diagnosis of DTD focuses on traumatization as the psychopathological trigger of mental disorders [94]. Several well-designed studies clearly demonstrated such correlations. Post-traumatic symptoms may occur together with other mental disorders. As many as 80% of PTSD patients meet the criteria for another disorder [95-98]. In an evaluation of the ‘Dunedin longitudinal study’, Koenen *et al*. [15] showed that all subjects meeting the criteria for PTSD in young adulthood had suffered from mental disorders at a young age. Conversely, other mental disorders may be present before PTSD or may develop after its occurrence [15,87,92]. In particular, victims of sequential traumatization have an inherently high risk of developing a complex syndrome of disorders that often go hand-in-hand with single symptoms of PTSD without fulfilling the complete clinical picture of PTSD [14]. In children and adolescents, comorbidities with ADHD, anxiety disorder, suicidal thoughts, and a trend towards affective disorders is highly prevalent [1,98].

*· Enable effective treatment*: By selectively treating trauma symptoms, patients can be stabilized, and concomitant illnesses (like anxiety disorder or depression) can be addressed. The effectiveness of therapeutic interventions in traumatized children and adolescents has been well documented in recent years [99-103]. Spinazzola *et al.*[104] pointed out that more attention should be given to naturalistic studies in inpatients suffering from psychosocial stress being at risk of suicide.

Patients with severe interpersonal traumatization in childhood are the hardest to treat and have the poorest prognosis. Treatment may be constrained by insufficient understanding of the underlying illness, and patients often cannot be reached by the psychosocial care system. Moreover, the degree of traumatization affects treatment success. Therefore, it is important to take the nature and severity of traumatic experiences into account when developing a treatment plan. With a more specific diagnosis, treatment options can be tailor-made.

*· Social and legal aspects*: Many victims of neglect, child abuse, and maltreatment live on the edge of society and depend on social services for most of their lives. Failures at school and in youth welfare institutions are common [105]. Clear definition of trauma-related symptoms could help to change attitudes towards delinquent or aggressive adolescents and facilitate the initiation of treatment [106].

Several studies have addressed the enormous healthcare costs arising from traumatization, such as medical treatment costs, early retirement, inability to work, need for social benefits, and even imprisonment [107,108]. If the consequences of childhood traumatization were officially recognized, patients would benefit from improved social acceptance of their difficulties. Moreover, inclusion of mental disorders arising from complex traumatization in the official diagnostic systems would assist patients in obtaining compensation and legal support (court, victim aid). Many traumatized patients develop chronic mental disorders with serious impairment of their working ability and social interactions. Early and effective intervention is necessary to help patients to maintain a normal life style.

#### Arguments against formalized DTD diagnostic criteria

The following arguments question the usefulness of including DTD as a distinct mental disorder in diagnostic systems:

*· Conflicting DSM and ICD diagnostic systems*: Formal DTD diagnostic criteria are thought to weaken the power of existing diagnostic systems, such as DSM-IV-TR and ICD-10. Both diagnostic systems were strictly designed to exclude any theory about the etiology of the mental disorders and confine themselves to a clear and operationalizable description of the symptoms and disorders. Since Axis V of the multiaxial diagnostic system covers psychosocial risk factors, aspects associated with chronic exposures to traumatic events are included in existing systems. In addition, critics claim that there is no clear distinction between symptoms and syndromes, and that DTD criteria overlap with those of some established and some discussed diagnoses. Many symptoms of borderline personality disorder or attachment disorder are included in the list of DTD symptoms, thus impeding the distinction between these disorders. Similarly, DTD criteria overlap with those of attachment disorders, conduct disorder, multiple complex development disorders (MCDD) [109] or the criteria for borderline disorder in childhood and adolescence [28]. Although, all of these diagnosis have a high prevalence among people with traumatic life events, problems with validity and reliability [110,111] and high comorbidities with other mental disorders. Some diagnosis like multiple complex trauma disorder and borderline personality disorder in childhood are not part of the diagnostic systems.

*· Monocausality*: concerning the diagnosis of DTD, monocausality is assumed, but this has not been proven [112]. DTD diagnosis favors a psychosocial explanation for the etiology of the disorders and neglects the biological explanations of the biopsychosocial model to understand the development of mental disorders. DTD is frequently manifested as a mixture of symptoms and syndromes, and a unidirectional relationship between traumatic experiences and the development of a confined syndrome remains is based on a widespread of actual research in the field of psycho traumatology. Moreover, genetic/biological causes of the symptom pattern may be ignored when diagnosing DTD. Critics of a formal DTD diagnosis point out that those similar symptoms may be present in individuals who did not have any traumatic experiences. In line with this, 20% to 30% of patients with borderline personality disorder, whose criteria are similar to those of complex PTSD, had not suffered from any traumatic experience [30]. By explaining complex symptom patterns by a single cause, other disorders that require treatment may remain untreated. Focussing on trauma etiology it might be possible that other comorbid diagnosis like ADHD will not be taken into account and missed to treat with evidenced based interventions. Furthermore, assumption of traumatization as the single cause of the disorder may result in too much importance being attached to identifying the causative traumatic experience, thus ignoring positive life experiences that would facilitate a resource-orientated therapeutic relationship, especially with the parents.

*· Selectivity:* Certain children who had been severely traumatized do not develop any mental disorder [113]. Of course this is a weak argument because skeptics can argue in the same way against the classic PTSD diagnosis.According to Malinosky-Rummell and Hansen, 80% of adults who had been physically abused during childhood showed no mental disorder in adulthood [114]. However, Collishaw *et al.*[115] found considerably weaker psychopathological resilience in a follow-up analysis of adults who had experienced maltreatment during childhood. Furthermore a study of the Dunedin birth cohort (in [15]) suggested that the risk of developing a mental disorder increases with repeated traumatization. Individuals who did not develop any symptoms were found to have good peer relations, success at school and work, and stable relationships. Current research into resilience increasingly focuses on dynamic factors, such as behavior and attitude, which enhance individual or familial resilience [113], and their correlation with genetic factors. Conversely, non-traumatized individuals may develop similar symptoms. The formal DTD criteria do not explain this phenomenon. In addition, there is a relatively high overlap with existing and well-established mental disorders (e.g., borderline disorder, attachment disorder with disinhibition, etc.).

*· Inverse correlation*: Diagnosing DTD implies that emotional dysregulation is caused by traumatic experiences but ignores the fact that the reverse relationship also exists. Emotional dysregulation is accompanied by a higher risk of traumatization. It is well established that subjects with impaired emotional control may adversely respond to environmental factors, thus reinforcing the present symptoms [116]. This correlation was described in the transactional model by Fruzzetti et al. [116]. Furthermore, children with externalizing disorders have a four times higher risk of being abused [117].

*· Age sensitivity*: Although the proposed diagnostic criteria are meant to take the age and developmental status of the patient into account, symptoms are not sufficiently stipulated age-sensitive. But of course this is a problem of every diagnosis in childhood and adolescence – regarding the actual debate among assessing symptoms of attention deficit and hyperactivity disorder ADHD in childhood, adolescence and adulthood [118]. Furthermore the criteria claim to be development-oriented, however they fail to specify the symptoms for different age groups. Thus, no distinction is being made between young children and adolescents with respect to emotional and physiological regulation. This is due to the limited knowledge about the course of trauma-related symptoms and the methodical problems in longitudinal studies to address the same construct in different age groups with other psychometric methods. Additionally clinical studies are limited by ethical restraints.

*· Treatment*: The main purpose of accurately diagnosing psychopathological conditions in children and adolescence is the endeavor to treat them effectively. Critics of the introduction of formal DTD diagnostic criteria argue that comorbidities may remain untreated because too much emphasis is placed on trauma-related aspects of the condition. This can provoke misinterpretations of biological symptoms with the consequence that effective psycho-pharmaceutical treatment options stay unused.

*· Disadvantages of trauma-focused diagnostic explorations:* For inexperienced professionals the concentration on trauma-related symptoms in the diagnostic process may result in a pressure to detect traumatic life events. This kind of exploration might have a negative influence on the therapeutic relationship, especially to parents of multi-problem families. It can be difficult to combine a trauma-focused exploration style with solution focused interventions. But without the development of a sustainable therapeutic relationship every treatment will fail. Another negative aspect of trauma-focused diagnostic exploration could be that patients will be pushed in an implicit or explicit way to remember or to talk about traumatic events. It is even possible that some trauma-focused exploration styles provoke false memories of biographical life events with several negative consequences [119].

## Summary

There is considerable controversy with respect to implementing formal DTD diagnostic criteria; based on existing empirical studies the correlation between traumatic experiences and related symptoms is not in question among experts. Studies focusing on the neurobiology of mental disorder in childhood have clearly identified traumatization as an important cause [120].

The current debate on the need for a formal definition of DTD criteria highlights the important role of traumatization and neglect in the development of complex psychopathological disorders that are difficult to treat. Awareness of long-term outcomes of child abuse and neglect may strengthen the acceptance of initiatives to protect children from maltreatment and improve attitudes towards ‘difficult’ adolescents who live at the edge of society. A better understanding of the effects of traumatization might lead to improved psychosocial treatment options for these children and adolescents and may help to prevent from participation restrictions in the society.

The arguments for and against implementing formal DTD diagnostic criteria are convincing, and the debate can only be resolved conclusively based on the emergence of new information. Sophisticated neurobiological and genetic studies are needed because traumatization is known to affect prenatal factors, such as endocrinological processes during and after pregnancy, or even genotype [121-124]. Moreover, longitudinal studies are necessary because DTD is not a static but a rather dynamic condition, undergoing changes in its manifestation over time. An innovative method using a developmental-heterotopic approach has been described by Fegert *et al.* and Schmid *et al.*[4,125,126].

In addition, clusters of mental disorders should be identified, and interaction of psychosocial and biological aspects in the development of these clusters should be addressed. Such an approach would help to explain the pervasive nature of trauma-related psychopathological disorders.

Trauma experts working in specialized institutions that deal exclusively with traumatized individuals tend to be the main supporters of a formal definition of DTD diagnostic criteria, while professionals working in the general clinical and psychiatric setting remain critical for the reasons stipulated above. Regardless of the outcome of the ongoing debate, treatment of severely traumatized children and adolescents should be improved substantially. Although trauma outpatient clinics offering symptom-specific treatment will be of help, general psychotherapeutic professionals also need to be trained in this area since many traumatized children are encountered in the clinical setting. Therapeutic concepts currently available for hospitalized patients are grossly inadequate to address the dramatic squeal in severely traumatized children. Trauma-specific concepts of outpatient treatment with possible inpatient interval treatment should be developed and implemented [101,127-129], taking the specific needs of children and adolescents into account as well as the need of their parents, foster parents or residential care staff. It is important to be able to combine both treatment needs: to maintain a “save place” and to have the possibility to do effective (prolonged) exposure therapy. For severely traumatized patients a combination of a skill training and trauma therapeutic exposure treatment is currently regarded to be the best approach [101,103] with the least drop-out rates. The trauma system therapy as a model of combined milieu therapeutic, systemic / family centered and psychotherapeutic intervention is a very promising and, as the first results show, successful treatment approach for children and adolescents suffering from complex trauma or developmental trauma disorder [130]. The psychotherapeutic skill training focuses on the capacities to cope with dissociation, emotion regulation problems, situations of extreme stress and tension as well as intrusions, disgust and social problems [101,103,127]. The additive skill training will help to overcome tension and dissociation during the exposure therapy and is a kind of precondition for exposure therapy with complex traumatized patients with fewer capacities to cope with stress, tension and dissociation [131]. The dialectical behavior therapy and their adaptions for adolescents [132,133] are the best evaluated treatment concepts to improve these skills. For such treatment concepts to be effective, specialized wards are needed, which will probably require inpatient treatment for a greater catchment area and build a network of outpatient therapists cooperating with this specialized ward.

As many severely traumatized children and adolescents cannot stay in their families of origin, psychiatric liaison services for adolescents in residential care institutions and youth welfare services should be implemented. These liaison services can help to reach more burdened children, reduce inpatient child- and adolescent psychiatric treatment days and improve continuity in residential and foster care placements [134]. Youth welfare concepts should be sensitized to trauma symptoms and try to promote and enhance resilience factors, self-efficacy and social and emotion-regulation skills [106]. In conclusion, the available arguments for and against the implementation formal diagnostic criteria for DTD cannot be appraised conclusively based on current research. The main advantage appears to be improved sensitization to trauma outcomes and more tailor-made treatment options, but this may also be achieved by a descriptive approach. A dimensional diagnostic system comprising the relevant domains, such as relationship / attachment representation, assessing interpersonal trust, emotion regulation, affinity to dissociation / sensual perception, and lacking expectation of self-efficacy, could also be envisaged. Specific symptom scales for emotion regulation, attachment/ interpersonal trust, self-efficacy and dissociation may be effective in predicting the outcome of psychotherapeutic treatment. These symptom scales may show relevant aspects of developmental psychopathology, can support the diagnostic process, and help to develop individualized treatment concepts with specific guidelines for the arrangement of the therapeutic alliance. Probably the sensitization to trauma symptoms and the interpersonal learning history of a patient can prevent drop-out and improve the therapeutic outcome.

## Competing interests

The authors declare that they have no competing interests.

## Authors’ contributions

JMF and FP contributed equally to this work. This paper is based on a former German publication by MS, JMF, FP. (2010) Traumaentwicklungsstörung: Pro und Contra. Kindheit & Entwicklung, 19 (1), 47–63. All authors read and approved the final manuscript.

## Authors’ information

Dr. Marc Schmid is chief psychologist at the department of child and adolescent psychiatry at the University Basel (Switzerland). Head of the center for the psychiatric and psychotherapeutic liaison services with youth welfare institutions and the EQUALS project.

Prof. Dr. Franz Petermann is Director of the center of rehabilitation and clinical psychology and professor for psychological diagnostics and intervention at the University Bremen (Germany).

Prof. Dr. Jörg M. Fegert is Medical Director of the department for child and adolescent psychiatry at the University of Ulm (Germany). Professor Fegert is member of diverse academic advisory boards of the German government among family affairs, research, child abuse and neglect.

## Pre-publication history

The pre-publication history for this paper can be accessed here:

http://www.biomedcentral.com/1471-244X/13/3/prepub

## References

[B1] Cloitre M Stolbach BC Herman JL Kolk BV Pynoos R Wang J Petkova E A developmental approach to complex PTSD: Childhood and adult cumulative trauma as predictors of symptom complexity J Trauma Stress 2009 22 5 399 408 10.1002/jts.20444 19795402

[B2] Terr LC Childhood traumas: an outline and overview Am J Psychiatry 1991 148 1 10 20 1824611 10.1176/ajp.148.1.10

[B3] van der Kolk BA Roth S Pelcovitz D Sunday S Spinazzola J Disorders of extreme stress: The empirical foundation of a complex adaptation to trauma J Trauma Stress 2005 18 5 389 399 10.1002/jts.20047 16281237

[B4] D'Andrea W Ford J Stolbach B Spinazzola J van der Kolk BA Understanding interpersonal trauma in children: why we need a developmentally appropriate trauma diagnosis Am J Orthopsychiatry 2012 82 2 187 200 10.1111/j.1939-0025.2012.01154.x 22506521

[B5] Euser EM van Ijzendoorn M Prinzie P Bakermans-Kranenburg MJ The Prevalence of Child Maltreatment in the Netherlands Child Maltreat 2010 15 1 5 17 10.1177/1077559509345904 19729577

[B6] Scheeringa MS Zeanah CH Myers L Putnam FW New findings on alternative criteria for PTSD in preschool children J Am Acad Child Adolesc Psychiatry 2003 42 5 561 570 10.1097/01.CHI.0000046822.95464.14 12707560

[B7] Simons M Herpertz-Dahlmann B Traumata und Traumafolgestörungen bei Kindern und Jugendlichen - eine kritische Übersicht zu Klassifkation und diagnostischen Kriterien Z Kinder Jugendpsychiatr Psychother 2008 36 3 151 161 10.1024/1422-4917.36.3.151 18622975

[B8] Finkelhor D Ormrod RK Turner HA Poly-victimization: a neglected component in child victimization Child Abuse Negl 2007 31 1 7 26 10.1016/j.chiabu.2006.06.008 17224181

[B9] Finkelhor D Ormrod RK Turner HA Lifetime assessment of poly-victimization in a national sample of children and youth Child Abuse Negl 2009 33 7 403 411 10.1016/j.chiabu.2008.09.012 19589596

[B10] Kienberger Jaudes P Mackey-Bilaver L Do chronic conditions increase young children's risk of being maltreated? Child Abuse Negl 2008 32 7 671 681 10.1016/j.chiabu.2007.08.007 18620753

[B11] Larson K Russ SA Crall JJ Halfon N Influence of multiple social risks on children's health Pediatrics 2008 121 2 337 344 10.1542/peds.2007-0447 18245425

[B12] Classen CC Palesh OG Aggarwal R Sexual revictimization: a review of the empirical literature Trauma Violence Abuse 2005 6 2 103 129 10.1177/1524838005275087 15753196

[B13] Widom CS Czaja SJ Dutton MA Childhood victimization and lifetime revictimization Child Abuse Negl 2008 32 8 785 796 10.1016/j.chiabu.2007.12.006 18760474 PMC2572709

[B14] Copeland WE Keeler G Angold A Costello EJ Traumatic events and posttraumatic stress in childhood Arch Gen Psychiatry 2007 64 5 577 584 10.1001/archpsyc.64.5.577 17485609

[B15] Koenen KC Moffitt TE Caspi A Gregory A Harrington H Poulton R The developmental mental-disorder histories of adults with posttraumatic stress disorder: a prospective longitudinal birth cohort study J Abnorm Psychol 2008 117 2 460 466 18489223 10.1037/0021-843X.117.2.460 PMC2666441

[B16] Pelcovitz D Kaplan SJ DeRosa RR Mandel FS Salzinger S Psychiatric disorders in adolescents exposed to domestic violence and physical abuse Am J Orthopsychiatry 2000 70 3 360 369 10953782 10.1037/h0087668

[B17] De Bellis MD Developmental traumatology: the psychobiological development of maltreated children and its implications for research, treatment, and policy Dev Psychopathol 2001 13 3 539 564 10.1017/S0954579401003078 11523847

[B18] Widom CS Posttraumatic stress disorder in abused and neglected children grown up Am J Psychiatry 1999 156 8 1223 1229 10450264 10.1176/ajp.156.8.1223

[B19] van der Kolk BA Developmental Trauma Disorder: Toward a rational diagnosis for children with complex trauma histories Psychiatr Ann 2005 35 5 401 408

[B20] VanderKolk BA Pynoos RS Cicchetti D Cloitre M D’Andrea W Ford JD Lieberman AF Putnam FW Saxe G Spinazzola J Stolbach BC Teicher M Proposal to include a developmental trauma disorder diagnosis for children and adolescents in DSM-V http://www.traumacenter.org/announcements/DTD_papers_Oct_09.pdf

[B21] De Bellis MD The psychobiology of neglect Child Maltreat 2005 10 2 150 172 10.1177/1077559505275116 15798010

[B22] Cicchetti D Toth SL Cicchetti D, Cohen DJ Development psychopathology and disorders of affect Developmental Psychopathology 1995 Wiley, New York 369 420

[B23] Pollak SD Sinha P Effects of early experience on children's recognition of facial displays of emotion Dev Psychol 2002 38 5 784 791 12220055 10.1037//0012-1649.38.5.784

[B24] Walden TA Smith MC Emotion regulation Motiv Emot 1997 21 1 7 25

[B25] Shields A Cicchetti D Reactive aggression among maltreated children: the contributions of attention and emotion dysregulation J Clin Child Psychol 1998 27 4 381 395 10.1207/s15374424jccp2704_2 9866075

[B26] Domes G Lischke A Berger C Grossmann A Hauenstein K Heinrichs M Herpertz SC Effects of intranasal oxytocin on emotional face processing in women Psychoneuroendocrinology 2010 35 1 83 93 10.1016/j.psyneuen.2009.06.016 19632787

[B27] Schore AN Schore AN Effect of early relational trauma on affect regulation: The development od borderline and antisocial personality disorders and a predisposition to violence Affect dysregulation and disorders of the self 2003 W.W. Norton, New York 266 306

[B28] Schmid M Schmeck K Petermann F Persönlichkeitsstörungen im Kindes- und Jugendalter? Kindheit und Entwicklung 2008 17 3 190 202 10.1026/0942-5403.17.3.190

[B29] Ebner-Priemer UW Kuo J Schlotz W Kleindienst N Rosenthal MZ Detterer L Linehan MM Bohus M Distress and affective dysregulation in patients with borderline personality disorder: a psychophysiological ambulatory monitoring study J Nerv Ment Dis 2008 196 4 314 320 10.1097/NMD.0b013e31816a493f 18414126

[B30] Ebner-Priemer UW Welch SS Grossman P Reisch T Linehan MM Bohus M Psychophysiological ambulatory assessment of affective dysregulation in borderline personality disorder Psychiatry Res 2007 150 3 265 275 10.1016/j.psychres.2006.04.014 17321599

[B31] Stiglmayr CE Ebner-Priemer UW Bretz J Behm R Mohse M Lammers CH Anghelescu IG Schmahl C Schlotz W Kleindienst N Dissociative symptoms are positively related to stress in borderline personality disorder Acta Psychiatr Scand 2008 117 2 139 147 18028248 10.1111/j.1600-0447.2007.01126.x

[B32] Lynch SM Forman E Mendelsohn M Herman J Attending to dissociation: assessing change in dissociation and predicting treatment outcome J Trauma Dissociation 2008 9 3 301 319 10.1080/15299730802139063 19042780

[B33] Merckelbach H Muris P The causal link between self-reported trauma and dissociation: a critical review Behav Res Ther 2001 39 3 245 254 10.1016/S0005-7967(99)00181-3 11227807

[B34] Zucker M Spinazzola J Blaustein M van der Kolk BA Dissociative symptomatology in posttraumatic stress disorder and disorders of extreme stress J Trauma Dissociation 2006 7 1 19 31 10.1300/J229v07n01_03 16618693

[B35] Brewin CR Andrews B Valentine JD Meta-analysis of risk factors for posttraumatic stress disorder in trauma-exposed adults J Consult Clin Psychol 2000 68 5 748 766 11068961 10.1037//0022-006x.68.5.748

[B36] Tuulikki Kultalahti T Rosner R Risikofaktoren der Posttraumatischen Belastungsstörung nach Trauma-Typ-I Kindheit und Entwicklung 2008 17 4 210 218 10.1026/0942-5403.17.4.210

[B37] Macfie J Cicchetti D Toth SL Dissociation in maltreated versus nonmaltreated preschool-aged children Child Abuse Negl 2001 25 9 1253 1267 10.1016/S0145-2134(01)00266-6 11700697

[B38] DiTomasso MJ Routh DK Recall of abuse in childhood and three measures of dissociation Child Abuse Negl 1993 17 4 477 485 10.1016/0145-2134(93)90022-W 8402250

[B39] Merckelbach H Muris P Rassin E Fantasy proneness and cognitive failures as correlates of dissociative experiences Personal Individ Differ 1999 26 5 961 967 10.1016/S0191-8869(98)00193-7

[B40] Sack M Boroske-Leiner K Lahmann C Association of nonsexual and sexual traumatizations with body image and psychosomatic symptoms in psychosomatic outpatients Gen Hosp Psychiatry 2010 32 3 315 320 10.1016/j.genhosppsych.2010.01.002 20430236

[B41] Schmid M In-Albon T Komplexe Traumatisierung und deren Auswirkungen auf die implizite und explizite Emotionsregulation Emotions regulation bei psychischen Erkrankungen im Kindes- und Jugendalter Kohlhammer, Stuttgart in press

[B42] Ludäscher P Bohus M Lieb K Philipsen A Jochims A Schmahl C Elevated pain thresholds correlate with dissociation and aversive arousal in patients with borderline personality disorder Psychiatry Res 2007 149 1–3 291 296 17126914 10.1016/j.psychres.2005.04.009

[B43] Klossika I Flor H Kamping S Bleichhardt G Trautmann N Treede RD Bohus M Schmahl C Emotional modulation of pain: a clinical perspective Pain 2006 124 3 264 268 10.1016/j.pain.2006.08.007 16934927

[B44] Maercker A Karl A Lifespan-developmental differences in physiologic reactivity to loud tones in trauma victims: a pilot study Psychol Rep 2003 93 3 Pt 1 941 948 14723466 10.2466/pr0.2003.93.3.941

[B45] Barker DJ Fetal nutrition and cardiovascular disease in later life Br Med Bull 1997 53 1 96 108 10.1093/oxfordjournals.bmb.a011609 9158287

[B46] Dobie DJ Kivlahan DR Maynard C Bush KR Davis TM Bradley KA Posttraumatic stress disorder in female veterans: association with self-reported health problems and functional impairment Arch Intern Med 2004 164 4 394 400 10.1001/archinte.164.4.394 14980990

[B47] Seng JS Graham-Bermann SA Clark MK McCarthy AM Ronis DL Posttraumatic stress disorder and physical comorbidity among female children and adolescents: results from service-use data Pediatrics 2005 116 6 e767 e776 10.1542/peds.2005-0608 16322133

[B48] Martin J Hiscock H Hardy P Davey B Wake M Adverse associations of infant and child sleep problems and parent health: an Australian population study Pediatrics 2007 119 5 947 955 10.1542/peds.2006-2569 17473096

[B49] Noll JG Trickett PK Susman EJ Putnam FW Sleep disturbances and childhood sexual abuse J Pediatr Psychol 2006 31 5 469 480 15958722 10.1093/jpepsy/jsj040

[B50] Yates TM Carlson EA Egeland B A prospective study of child maltreatment and self-injurious behavior in a community sample Dev Psychopathol 2008 20 2 651 671 18423099 10.1017/S0954579408000321

[B51] Bruffaerts R Demyttenaere K Borges G Haro JM Chiu WT Hwang I Karam EG Kessler RC Sampson N Alonso J Childhood adversities as risk factors for onset and persistence of suicidal behaviour Br J Psychiatry 2010 197 1 20 27 10.1192/bjp.bp.109.074716 20592429 PMC2894980

[B52] Plener PL Singer H Goldbeck L Traumatic events and suicidally in a German adolescent community sample J Trauma Stress 2011 24 1 121 124 10.1002/jts.20598 21351171

[B53] van der Kolk BA Perry JC Herman JL Childhood origins of self-destructive behavior Am J Psychiatry 1991 148 12 1665 1671 1957928 10.1176/ajp.148.12.1665

[B54] Klonsky ED Moyer A Childhood sexual abuse and non-suicidal self-injury: meta-analysis Br J Psychiatry 2008 192 3 166 170 10.1192/bjp.bp.106.030650 18310572

[B55] Weierich MR Nock MK Posttraumatic stress symptoms mediate the relation between childhood sexual abuse and nonsuicidal self-injury J Consult Clin Psychol 2008 76 1 39 44 18229981 10.1037/0022-006X.76.1.39

[B56] Lang CM Sharma-Patel K The relation between childhood maltreatment and self-injury: a review of the literature on conceptualization and intervention Trauma Violence Abuse 2011 12 1 23 37 10.1177/1524838010386975 21288933

[B57] Afifi TO Boman J Fleisher W Sareen J The relationship between child abuse, parental divorce, and lifetime mental disorders and suicidality in a nationally representative adult sample Child Abuse Negl 2009 33 3 139 147 10.1016/j.chiabu.2008.12.009 19327835

[B58] Klonsky ED The functions of deliberate self-injury: a review of the evidence Clin Psychol Rev 2007 27 2 226 239 10.1016/j.cpr.2006.08.002 17014942

[B59] Corrigan FM Fisher JJ Nutt DJ Autonomic dysregulation and the Window of Tolerance model of the effects of complex emotional trauma J Psychopharmacol 2011 25 1 17 25 10.1177/0269881109354930 20093318

[B60] Glassman LH Weierich MR Hooley JM Deliberto TL Nock MK Child maltreatment, non-suicidal self-injury, and the mediating role of self-criticism Behavior Research and Therapy 2007 45 10 2483 2490 10.1016/j.brat.2007.04.002 PMC2034449 17531192

[B61] Elkovitch N Latzman RD Hansen DJ Flood MF Understanding child sexual behavior problems: a developmental psychopathology framework Clin Psychol Rev 2009 29 7 586 598 10.1016/j.cpr.2009.06.006 19664867

[B62] Wells RD McCann J Adams J Voris J Ensign J Emotional, behavioral, and physical symptoms reported by parents of sexually abused, nonabused, and allegedly abused prepubescent females Child Abuse Negl 1995 19 2 155 163 10.1016/0145-2134(94)00113-9 7780778

[B63] Shafii T Rivara FP Wang J Jurkovich GJ Screening Adolescent Patients Admitted to the Trauma Service for High-Risk Behaviors: Who Is Responsible? J Trauma 2009 67 6 1288 1292 10.1097/TA.0b013e3181847e8e 19779312

[B64] Blomeyer D Treutlein J Esser G Schmidt MH Schumann G Laucht M Interaction between CRHR1 gene and stressful life events predicts adolescent heavy alcohol use Biol Psychiatry 2008 63 2 146 151 10.1016/j.biopsych.2007.04.026 17597588

[B65] Beckett C Maughan B Rutter M Castle J Colvert E Groothues C Hawkins A Kreppner J O'Connor TG Stevens S Scholastic attainment following severe early institutional deprivation: a study of children adopted from Romania J Abnorm Child Psychol 2007 35 6 1063 1073 10.1007/s10802-007-9155-y 17643189

[B66] Colvert E Rutter M Beckett C Castle J Groothues C Hawkins A Kreppner J O'Connor TG Stevens S Sonuga-Barke EJ Emotional difficulties in early adolescence following severe early deprivation: Findings from the English and Romanian adoptees study Dev Psychopathol 2008 20 2 547 567 18423094 10.1017/S0954579408000278

[B67] Rutter M Colvert E Kreppner J Beckett C Castle J Groothues C Hawkins A O'Connor TG Stevens SE Sonuga-Barke EJ Early adolescent outcomes for institutionally-deprived and non-deprived adoptees. I: disinhibited attachment J Child Psychol Psychiatry 2007 48 1 17 30 10.1111/j.1469-7610.2006.01688.x 17244267

[B68] Beers SR De Bellis MD Neuropsychological function in children with maltreatment-related posttraumatic stress disorder Am J Psychiatry 2002 159 3 483 486 10.1176/appi.ajp.159.3.483 11870018

[B69] Navalta CP Polcari A Webster DM Boghossian A Teicher MH Effects of childhood sexual abuse on neuropsychological and cognitive function in college women J Neuropsychiatry Clin Neurosci 2006 18 1 45 53 10.1176/appi.neuropsych.18.1.45 16525070

[B70] Savitz J Jansen P The Stroop Color-Word Interference Test as an indicator of ADHD in poor readers The Journal of Genetic Psychology: Research and Theory on Human Development 2003 164 3 319 333 10.1080/00221320309597986 14521215

[B71] Endo T Sugiyama T Someya T Attention-deficit/hyperactivity disorder and dissociative disorder among abused children Psychiatry Clin Neurosci 2006 60 4 434 438 10.1111/j.1440-1819.2006.01528.x 16884444

[B72] Hipwell AE Goossens FA Melhuish EC Kumar R Severe maternal psychopathology and infant-mother attachment Dev Psychopathol 2000 12 2 157 175 10.1017/S0954579400002030 10847622

[B73] Muller RT Sicoli LA Lemieux KE Relationship between attachment style and posttraumatic stress symptomatology among adults who report the experience of childhood abuse J Trauma Stress 2000 13 2 321 332 10.1023/A:1007752719557 10838678

[B74] Van Ijzendoorn MH Schuengel C Bakermans-Kranenburg MJ Disorganized attachment in early childhood: Meta-analysis of precursors, concomitants, and sequelae Dev Psychopathol 1999 11 2 225 249 10.1017/S0954579499002035 16506532

[B75] Kim J Cicchetti D A longitudinal study of child maltreatment, mother-child relationship quality and maladjustment: the role of self-esteem and social competence J Abnorm Child Psychol 2004 32 4 341 354 15305541 10.1023/b:jacp.0000030289.17006.5a

[B76] Weinfield NS Sroufe LA Egeland B Attachment from infancy to early adulthood in a high-risk sample: Continuity, discontinuity, and their correlates Child Dev 2000 71 3 695 702 10.1111/1467-8624.00178 10953936

[B77] O'Connor TG Rutter M Attachment disorder behavior following early severe deprivation: extension and longitudinal follow-up. English and Romanian Adoptees Study Team J Am Acad Child Adolesc Psychiatry 2000 39 6 703 712 10.1097/00004583-200006000-00008 10846304

[B78] Elliott GC Cunningham SM Linder M Colangelo M Gross M Child Physical Abuse and Self-Perceived Social Isolation Among Adolescents J Interpers Violence 2005 20 12 1663 1684 10.1177/0886260505281439 16246923

[B79] Skodol AE Bender DS Pagano ME Shea MT Yen S Sanislow CA Grilo CM Daversa MT Stout RL Zanarini MC Positive childhood experiences: resilience and recovery from personality disorder in early adulthood J Clin Psychiatry 2007 68 7 1102 1108 10.4088/JCP.v68n0719 17685749 PMC2705622

[B80] Dodge KA Schwartz D Stoff DM, Breiling J, Maser JD Social information-processing mechanisms in aggressive behaviour Handbook of antisocial behavior 1997 Wiley, New York 171 180

[B81] Pollak SD Tolley-Schell S Selective attention to facial emotion in physically abused children J Abnorm Psychol 2003 112 3 323 338 12943012 10.1037/0021-843x.112.3.323

[B82] Cullerton-Sen C Cassidy AR Murray-Close D Cicchetti D Crick NR Rogosch FA Childhood maltreatment and the development of relational and physical aggression: the importance of a gender-informed approach Child Dev 2008 79 6 1736 1751 10.1111/j.1467-8624.2008.01222.x 19037946 PMC3397662

[B83] Ford JD Fraleigh LA Connor DF Child abuse and aggression among seriously emotionally disturbed children J Clin Child Adolesc Psychol 2010 39 1 25 34 20390796 10.1080/15374410903401104

[B84] Maughan A Cicchetti D Impact of child maltreatment and interadult violence on children's emotion regulation abilities and socioemotional adjustment Child Dev 2002 73 5 1525 1542 10.1111/1467-8624.00488 12361317

[B85] Wyatt GE Newcomb M Internal and external mediators of women's sexual abuse in childhood J Consult Clin Psychol 1990 58 6 758 767 2292625 10.1037//0022-006x.58.6.758

[B86] Tomoda A Navalta CP Polcari A Sadato N Teicher MH Childhood sexual abuse is associated with reduced gray matter volume in visual cortex of young women Biol Psychiatry 2009 66 7 642 648 10.1016/j.biopsych.2009.04.021 19560122 PMC4277202

[B87] Copeland WE Shanahan L Costello EJ Angold A Childhood and adolescent psychiatric disorders as predictors of young adult disorders Arch Gen Psychiatry 2009 66 7 764 772 10.1001/archgenpsychiatry.2009.85 19581568 PMC2891142

[B88] Kim J Cicchetti D Social self-efficacy and behavior problems in maltreated and nonmaltreated children J Clin Child Adolesc Psychol 2003 32 1 106 117 12573936 10.1207/S15374424JCCP3201_10

[B89] Pears KC Fisher PA Emotion understanding and theory of mind among maltreated children in foster care: evidence of deficits Dev Psychopathol 2005 17 1 47 65 15971759 10.1017/s0954579405050030

[B90] Pears KC Moses LJ Demographics, parenting, and theory of mind in preschool children Soc Dev 2003 12 1 1 20 10.1111/1467-9507.00219

[B91] Fonagy P Gergely G Jurist EL Target M Affect regulation, mentalization, and the development of the self 2002 Other Press, New York

[B92] Kim-Cohen J Caspi A Moffitt TE Harrington H Milne BJ Poulton R Prior juvenile diagnoses in adults with mental disorder: developmental follow-back of a prospective-longitudinal cohort Arch Gen Psychiatry 2003 60 7 709 717 10.1001/archpsyc.60.7.709 12860775

[B93] Perkonigg A Pfister H Stein MB Hofler M Lieb R Maercker A Wittchen HU Longitudinal course of posttraumatic stress disorder and posttraumatic stress disorder symptoms in a community sample of adolescents and young adults Am J Psychiatry 2005 162 7 1320 1327 10.1176/appi.ajp.162.7.1320 15994715

[B94] Brady KT Killeen TK Brewerton T Lucerini S Comorbidity of psychiatric disorders and posttraumatic stress disorder J Clin Psychiatry 2000 61 Suppl 7 22 32 10795606

[B95] Essau CA Conradt J Petermann F Häufigkeit der Posttraumatischen Belastungsstörung bei Jugendlichen: Ergebnisse der Bremer Jugendstudie Z Kinder Jugendpsychiatr Psychother 1999 27 1 37 45 10.1024//1422-4917.27.1.37 10096158

[B96] McFarlane AC Posttraumatic stress disorder: a model of the longitudinal course and the role of risk factors J Clin Psychiatry 2000 61 5 15 20 discussion 21–23 10761675

[B97] Ross CA The trauma model: a solution to the problem of comorbidity in psychiatry 2000 Manitou Communications, Richardson

[B98] Famularo R Fenton T Kinscherff R Augustyn M Psychiatric comorbidity in childhood post traumatic stress disorder Child Abuse Negl 1996 20 10 953 961 10.1016/0145-2134(96)00084-1 8902292

[B99] Rodenburg R Benjamin A de Roos C Meijer AM Stams GJ Efficacy of EMDR in children: a meta-analysis Clin Psychol Rev 2009 29 7 599 606 10.1016/j.cpr.2009.06.008 19616353

[B100] Cohen JA Deblinger E Mannarino AP Steer RA A multisite, randomized controlled trial for children with sexual abuse-related PTSD symptoms J Am Acad Child Adolesc Psychiatry 2004 43 4 393 402 10.1097/00004583-200404000-00005 15187799 PMC1201422

[B101] Cohen JA Mannarino AP Deblinger E Treating trauma and traumatic grief in children and adolescents 2006 Guilford Press, New York

[B102] Deblinger E Mannarino AP Cohen JA Steer RA A Follow-up Study of a Multisite, Randomized, Controlled Trial for Children With Sexual Abuse-Related PTSD Symptoms J Am Acad Child Adolesc Psychiatry 2006 45 12 1474 1484 10.1097/01.chi.0000240839.56114.bb 17135993

[B103] Cloitre M Stovall-McClough KC Nooner K Zorbas P Cherry S Jackson CL Gan W Petkova E Treatment for PTSD Related to Childhood Abuse: A Randomized Controlled Trial Am J Psychiatry 2010 167 8 915 924 10.1176/appi.ajp.2010.09081247 20595411

[B104] Spinazzola J Blaustein M van der Kolk BA Posttraumatic stress disorder treatment outcome research: The study of unrepresentative samples? J Trauma Stress 2005 18 5 425 436 10.1002/jts.20050 16281240

[B105] Schmid M Goldbeck L Nuetzel J Fegert JM Prevalence of mental disorders among adolescents in German youth welfare institutions Child and Adolescent Psychiatry and Mental Health 2008 2 1 2 10.1186/1753-2000-2-2 18226213 PMC2262059

[B106] Schmid M Entwicklungspsychopathologische Grundlagen einer Traumapädagogik Trauma & Gewalt 2008 2 4 288 309

[B107] Solomon SD Davidson JR Trauma: prevalence, impairment, service use, and cost J Clin Psychiatry 1997 58 Suppl 9 5 11 9329445

[B108] Kessler RC Posttraumatic stress disorder: the burden to the individual and to society J Clin Psychiatry 2000 61 5 4 12 discussion 13–14 10761674

[B109] Ad-Dab'bagh Y Greenfield B Multiple complex developmental disorder: the "multiple and complex" evolution of the "childhood borderline syndrome" construct J Am Acad Child Adolesc Psychiatry 2001 40 8 954 964 10.1097/00004583-200108000-00018 11501696

[B110] Moffitt TE Arseneault L Jaffee SR Kim-Cohen J Koenen KC Odgers CL Slutske WS Viding E Research review: DSM-V conduct disorder: research needs for an evidence base J Child Psychol Psychiatry 2008 49 1 3 33 10.1111/j.1469-7610.2007.01823.x 18181878 PMC2822647

[B111] Minnis H Marwick H Arthur J McLaughlin A Reactive attachment disorder–a theoretical model beyond attachment Eur Child Adolesc Psychiatry 2006 15 6 336 342 10.1007/s00787-006-0539-2 16685475

[B112] Schweiger U Sipos V Hohagen F Kritische Überlegungen zum Begriff der "komplexen posttraumatischen Belastungsstörung" Der Nervenarzt 2005 76 3 344 346 author reply 346–347 15892192 10.1007/s00115-004-1748-x

[B113] Luthar S Resilience and Vulnerability 2003 Cambridge University Press, Cambridge

[B114] Malinosky-Rummell R Hansen D Long-term consequences of childhood physical abuse Psychol Bull 1993 114 1 68 79 8346329 10.1037/0033-2909.114.1.68

[B115] Collishaw S Pickles A Messer J Rutter M Shearer C Maughan B Resilience to adult psychopathology following childhood maltreatment: evidence from a community sample Child Abuse Negl 2007 31 3 211 229 10.1016/j.chiabu.2007.02.004 17399786

[B116] Fruzzetti AE Shenk C Hoffman PD Family interaction and the development of borderline personality disorder: A transactional model Dev Psychopathol 2005 17 4 1007 1030 16613428 10.1017/s0954579405050479

[B117] Ouyang L Fang X Mercy J Perou R Grosse SD Attention-deficit/hyperactivity disorder symptoms and child maltreatment: a population-based study J Pediatr 2008 153 6 851 856 10.1016/j.jpeds.2008.06.002 18619612

[B118] Matte B Rohde LA Grevet EH ADHD in adults: a concept in evolution Atten Defic Hyperact Disord 2012 4 2 53 62 10.1007/s12402-012-0077-3 22588789

[B119] Jelinek L Hottenrott B Randjbar S Peters MJ Moritz S Visual false memories in post-traumatic stress disorder (PTSD) J Behav Ther Exp Psychiatry 2009 40 2 374 383 10.1016/j.jbtep.2009.02.003 19303587

[B120] Murray-Close D Han G Cicchetti D Crick NR Rogosch FA Neuroendocrine regulation and physical and relational aggression: the moderating roles of child maltreatment and gender Dev Psychol 2008 44 4 1160 1176 18605842 10.1037/a0012564 PMC2515713

[B121] Caspi A Moffitt TE Gene-environment interactions in psychiatry: joining forces with neuroscience Nat Rev Neurosci 2006 7 7 583 590 16791147 10.1038/nrn1925

[B122] Radtke KM Ruf M Gunter HM Dohrmann K Schauer M Meyer A Elbert T Transgenerational impact of intimate partner violence on methylation in the promoter of the glucocorticoid receptor Translational Psychiatry 2011 1 e21 10.1038/tp.2011.21 22832523 PMC3309516

[B123] Yehuda R Bierer LM The relevance of epigenetics to PTSD: Implications for the DSM-V J Trauma Stress 2009 22 5 427 434 10.1002/jts.20448 19813242 PMC2891396

[B124] Kim-Cohen J Caspi A Taylor A Williams B Newcombe R Craig IW Moffitt TE MAOA, maltreatment, and gene-environment interaction predicting children's mental health: new evidence and a meta-analysis Mol Psychiatry 2006 11 10 903 913 10.1038/sj.mp.4001851 16801953

[B125] Fegert JM Spröber N Streeck-Fischer A Freyberger HJ "Adoleszenzkrisen" aus entwicklungspsychologischer und psychiatrischer Sicht Psychodyn Psychother 2010 9 1 2 13

[B126] Schmid M Fegert JM Petermann F Traumaentwicklungsstörung: Pro und Contra Kindheit und Entwicklung 2010 19 1 47 63 10.1026/0942-5403/a000008

[B127] Schmid M Goldbeck L Kognitiv verhaltenstherapeutische Ansätze bei komplex traumatisierten Jugendlichen Prax Kinderpsychol Kinderpsychiatr 2010 59 6 453 476 20795522 10.13109/prkk.2010.59.6.453

[B128] Sachsse U Vogel C Leichsenring F Results of psychodynamically oriented trauma-focused inpatient treatment for women with complex posttraumatic stress disorder (PTSD) and borderline personality disorder (BPD) Bull Menninger Clin 2006 70 2 125 144 10.1521/bumc.2006.70.2.125 16753036

[B129] Steil R Dyer A Priebe K Kleindienst N Bohus M Dialectical behavior therapy for posttraumatic stress disorder related to childhood sexual abuse: a pilot study of an intensive residential treatment program J Trauma Stress 2011 24 1 102 106 10.1002/jts.20617 21351167

[B130] Ellis B Fogler J Hansen S Forbes P Navalta CP Saxe G Trauma systems therapy: 15-month outcomes and the importance of effecting environmental change Psychological Trauma: Theory, Research, Practice, and Policy Aug 2011 (Pagination):No Pagination Specified

[B131] Priebe K Steil R Kleindienst N Dyer AS Krueger A Bohus M Psychotherapie der Posttraumatischen Belastungsstörung nach sexuellem Missbrauch: Ein Überblick über die Datenlage Psychother Psychosom Med Psychol 2012 62 1 5 17 22271171 10.1055/s-0031-1295482

[B132] Linehan MM Cognitive-behavioral treatment of borderline personality disorder 1993 Guilford Press, New York

[B133] Miller AL Rathus JH Linehan MM Dialectical behavior therapy with suicidal adolescents 2006 Guilford Press, New York

[B134] Besier T Fegert JM Goldbeck L Evaluation of psychiatric liaison-services for adolescents in residential group homes Eur Psychiatry 2009 24 7 483 489 10.1016/j.eurpsy.2009.02.006 19553090

